# Comparison of laboratory indices of non-alcoholic fatty liver disease for the detection of incipient kidney dysfunction

**DOI:** 10.7717/peerj.6524

**Published:** 2019-03-08

**Authors:** Jong Wook Choi, Chang Hwa Lee, Joon-Sung Park

**Affiliations:** 1 Department of Internal Medicine, Konkuk University Chungju Hospital, Chungju, Republic of Korea; 2 Department of Internal Medicine, Hanyang University College of Medicine, Seoul, Republic of Korea

**Keywords:** Fibrosis-4, Non-alcoholic fatty liver disease, Chronic kidney disease

## Abstract

Non-alcoholic fatty liver disease (NAFLD) is closely linked to insulin resistance and related adverse health outcomes. We investigated the non-invasive index of NAFLD that has the best performance in estimating the renal manifestations of metabolic disturbances. This nation-wide, cross-sectional study included 11,836 subjects, using various non-invasive assessments comprising routinely measured clinical and laboratory variables. The subjects were native Koreans aged 20 years or older and had no diabetes, history of liver or kidney disease. All participants were divided into quintiles according to their fibrosis-4 (FIB-4) results. Participants in the highest quintile were more hypertensive and obese with greater glycemic exposure, poor lipid profiles, and impaired kidney function, than those in the other quintiles. Multiple logistic regression, adjusted for age, sex, smoking, systolic blood pressure, white blood cell, platelet, fasting plasma glucose, and triglyceride, demonstrated that FIB-4, the hepatic steatosis index, the aspartate aminotransferase/alanine aminotransferase (AST/ALT) ratio, Gholam’s model for non-alcoholic steatohepatitis, and the BARD score were independently associated with kidney dysfunction. ROC curve analysis revealed that FIB-4 (AUC = 0.6227, 95% CI [0.5929–0.6526], *p* = 0.0258) was the most precise in predicting kidney dysfunction. Our findings suggest that FIB-4 may be a favorable screening tool for the renal manifestation of hepatic metabolic disturbances.

## Introduction

Non-alcoholic fatty liver disease (NAFLD) is described as a clinico-histopathological entity encompassing a spectrum of chronic liver diseases ranging from asymptomatic hepatic fat accumulation to advanced liver diseases with clinical features that resemble those of alcoholic liver disease, in the absence of excessive alcohol consumption (Choi et al., 2017; Suzuki et al., 2005; Williams et al., 2011). Emerging evidence indicates that dysfunctional hepatic fat accumulation and its related local inflammation (hepatic necro-inflammation), together with insulin resistance, not only increase liver-related morbidity and mortality, but also cause extrahepatic manifestation that affects distant organ dysfunction, such as cardiovascular disease (CVD) and chronic kidney disease (CKD) (Bae et al., 2010; Choi et al., 2009; Kim et al., 2013; Targher & Byrne, 2017). The detection of a subject at risk of NAFLD is critical to minimize these irreversible consequences.

The kidney is a well-known target organ of systemic inflammation related to insulin resistance (De Cosmo et al., 2013). Previous clinical and experimental studies have demonstrated that the increased release of multiple pro-inflammatory mediators from dysfunctional hepatic adipose tissues can favor the initiation of low-grade systemic inflammation, atherogenic dyslipidemia, hypercoagulability, hypofibrinolysis, and increased blood pressure (BP); such pathologic reactions can contribute to vascular endothelial dysfunction and may be a major cause of the functional and structural derangement of various target organs (Byrne & Targher, 2015; Targher, Chonchol & Byrne, 2014). Despite the increasing evidence linking NAFLD and low-grade systemic inflammation, there is limited evidence of the negative impact of hepatic necro-inflammation on the progression to overt kidney disease.

For the assessment of pathologic changes related to NAFLD, liver biopsy is currently the gold standard (Rockey et al., 2009). Owing to its invasiveness, sampling variability, economic problems, and patient’s burden, it is frequently unfeasible for routine clinical practices (Machado & Cortez-Pinto, 2013; Poynard et al., 2004; Sanyal et al., 2011). To overcome such limitations, several alternative, non-invasive strategies based on standard laboratory tests and anthropometric parameters were recently introduced to assess hepatic necro-inflammation and its related liver fibrosis in patients with presumed NAFLD (Forns et al., 2002; Gholam et al., 2007; Lee et al., 2010; Raszeja-Wyszomirska et al., 2010; Wang et al., 2015). However, there is still controversy over the association between biomarker-based diagnosis of steatohepatitis and low-grade systemic inflammation. Furthermore, these biomarker-based indices were not fully validated as a useful tool to assess the extra-hepatic effects of NAFLD on the kidney, especially in the general population. To solve this problem, we designed this nation-wide, population-based study to find a possible association between non-invasive indices for NAFLD and impaired kidney function and compare their discriminative power in predicting kidney disease.

## Methods

### Study population

All data were obtained in the Korean National Health and Nutrition Examination Survey (KNHANES) conducted by the Korea Centers for Disease Control and Prevention (KCDC) among Korean non-institutionalized population. All the participants in the study were provided written informed consent at study entry. Their records were properly anonymized before analysis except for the date of the research. The Institutional Review Board (IRB) of the KCDC approved the study protocol (IRB: 2011-02CON-06-C, 2012-01EXP-01-2C, 2013-07CON-03-4C, 2013-12EXP-03-5C). The study was undertaken in accordance with the Helsinki Declaration of 1975.

A total of 35,954 individuals had participated in the KNHANES VI-IV. The exclusion criteria were: incomplete anthropometric or laboratory data; age < 20 years; pregnancy; a history of medical problems, such as kidney and/or liver disease; and missing results of fibrosis-4 (FIB-4).

Because our receiver operating characteristic (ROC) curves analysis demonstrated that FIB-4 was the most precise in estimating the extra-hepatic manifestation of hepatic necro-inflammation on the kidney function, we classified the final 11,836 participants, and divided them into quintiles according to their FIB-4 results, stratified by sex (Fig. S1).

### Anthropometric and clinical measurements

Well-trained examiners conducted anthropometric measurements. Participants wore a lightweight gown or underwear. Height measurement was done to the nearest 0.1 cm with a mobile stadiometer (Seriter, Bismarck, ND, USA). Weight was calibrated to the nearest 0.1 kg with a balance-beam scale (Giant-150N; Hana, Seoul, Korea). Measurement of waist circumference was made with a flexible tape at the narrowest spot, midway between the lowest costal border and the highest point of the iliac crest at the end of gentle expiration. Body mass index was calculated as a person’s weight (kilograms) divided by height squared (meters).

Three consecutive BP measurements were obtained using an auscultatory mercury sphygmomanometer (Baumanometer; Baum, Copiague, NY, USA) while in a sitting position, following a 5-minute rest period. The mean values of the three consecutive systolic and diastolic BP readings were used in this analysis.

### Laboratory tests

Venous blood collection were carried out after 8 h of overnight fasting. Fasting plasma concentrations of glucose, triglyceride (TG), high-density lipoprotein cholesterol, and low-density lipoprotein cholesterol were determined by a Hitachi Automatic Analyzer 7600 (Hitachi, Tokyo, Japan). Glycated hemoglobin levels were determined by high-pressure liquid chromatography with an analyzer HLC-723G7 (Tosoh Corporation, Tokyo, Japan). Serum creatinine (Cr) levels were measured colorimetrically (Hitachi 7600 automatic analyzer, Hitachi, Tokyo, Japan) and estimated glomerular filtration rate (eGFR) was measured using the Chronic Kidney Disease Epidemiology Collaboration equation (CKD-EPI) (Levey et al., 2009). To obtain the Urine albumin/Cr ratio (UACR), urine albumin was measured in spot urine by the immunoturbidimetric method and urinary Cr was measured using the colorimetric method.

Hepatic necro-inflammation and related liver fibrosis were estimated based on 11 different non-invasive scoring systems including Gholam’s model, BARD scores, and FIB-4 using routinely measured laboratory test and anthropometric parameters (Table S1).

### Definition

According to KDIGO 2012 Clinical Practice Guideline (Stevens & Levin, 2013), albuminuria was defined as UACR of 30 mg/g creatinine or more and participants with CKD was defined as those with either eGFR below 60 mL/min/1.73 m^2^ or albuminuria.

### Statistical analysis

All data, including social and demographic information, clinical findings, medical conditions, anthropometric features, and laboratory results, were presented as mean ± SE or frequencies (and proportions). Sampling weights were used to analyze multi-stage stratified sampling data. A Kolmogorov–Smirnov test was used to test for normality of the distribution. If the original data do not follow a Gaussian distribution, the natural logarithmic (Ln) transformation was applied to make the distribution more normal. Quantitative variables were compared by the generalized linear models. A Chi-square test was used to compare proportions for categorical variables. The relationship between conventional predictor variables and the non-invasive NAFLD indices was assessed by linear regression analysis. The odds ratios (ORs) and 95% confidence intervals (CIs) were calculated by multiple logistic regression models based on the presence of CKD (case vs. control). ROC curve analysis was performed to compare the diagnostic performance of most candidate non-invasive indices of NAFLD on kidney dysfunction and nonparametric methods previously described by DeLong, DeLong & Clarke-Pearson (1988) were used for comparing the areas under the ROC curves (AUCs).

A two-sided *p* <0.05 was regarded as statistically significant. Statistical analysis Software (SAS) version 9.4 (SAS Institute Inc, Cary, NC, USA) was used for all of the analyses.

## Results

### Baseline characteristics

The participants (*n* = 11,836) comprised 4,893 men and 6,943 women with a mean age of 44.4 ± 14.8 years. They were divided into five groups according to their FIB-4 results and were categorized by sex. Participants in the highest FIB-4 quintile were older and more obese than participants in the other quintiles. They had a tendency to have high systolic and diastolic BP, increased glycemic exposure, and poor lipid profiles. The other demographic data and clinical characteristics are presented in Table 1.

**Table 1:** General characteristics grouped according to fibrosis-4 (FIB-4).

	Quintile 1	Quintile 2	Quintile 3	Quintile 4	Quintile 5
FIB-4 in male participants	≥0.12, ≤0.50	>0.50, ≤0.69	>0.69, ≤0.92	>0.92, ≤1.30	>1.30, ≤11.23
FIB-4 in female participants	≥0.18, ≤0.50	>0.50, ≤0.66	>0.66, ≤0.85	>0.85, ≤1.16	>1.16, ≤10.02
Variables	(*n* = 2, 366)	(*n* = 2, 367)	(*n* = 2, 368)	(*n* = 2, 367)	(*n* = 2, 368)
Age (year)	22.7 ± 0.2	35.7 ± 0.2	43.7 ± 0.2	51.8 ± 0.2	62.8 ± 0.2
Sex (% male)	961 (41)	962 (41)	962 (41)	962 (41)	962 (41)
Current smoker (%)	594 (25)	569 (24)	501 (21)	436 (18)	341 (14)
Systolic BP (mmHg)	109.9 ± 0.2	112.4 ± 0.3	115.1 ± 0.3	119.2 ± 0.3	124.2 ± 0.3
Diastolic BP (mmHg)	70.5 ± 0.2	75.2 ± 0.2	76.7 ± 0.2	77.5 ± 0.2	75.6 ± 0.2
Body mass index (kg/m^2^)	22.5 ± 0.1	23.7 ± 0.1	23.7 ± 0.1	23.8 ± 0.1	23.8 ± 0.1
Waist circumference (cm)	75.7 ± 0.2	80.3 ± 0.2	80.8 ± 0.2	81.6 ± 0.2	82.2 ± 0.2
White blood cell (10^9^/L)	6.47 ± 0.03	6.37 ± 0.04	6.21 ± 0.03	6.04 ± 0.03	5.71 ± 0.02
Hemoglobin (g/dL)	14.33 ± 0.03	14.33 ± 0.04	14.28 ± 0.03	14.26 ± 0.03	13.96 ± 0.02
Platelet (10^3^/µL)	285.9 ± 0.9	267.8 ± 1.1	260.9 ± 1.1	246.7 ± 0.9	215.6 ± 0.8
eGFR^a^ (mL/min/1.73 m^2^)	118.9 ± 0.3	104.3 ± 0.3	98.1 ± 0.3	92.4 ± 0.3	84.3 ± 0.2
Fasting plasma glucose (mg/dL)	91.0 ± 0.2	94.3 ± 0.3	97.9 ± 0.4	101.6 ± 0.5	102.3 ± 0.4
Hemoglobin A1c (%)	5.48 ± 0.01	5.58 ± 0.01	5.72 ± 0.01	5.86 ± 0.02	5.94 ± 0.01
Aspartate aminotransferase (U/L)	18.3 ± 0.1	19.7 ± 0.2	21.0 ± 0.2	22.3 ± 0.2	28.2 ± 0.3
Alanine aminotransferase (U/L)	18.7 ± 0.3	22.0 ± 0.5	22.1 ± 0.4	21.3 ± 0.3	24.0 ± 0.5
*γ*-Glutamyl transferase (U/L)^a^	22.0 ± 0.6	31.7 ± 1.5	35.2 ± 2.1	33.5 ± 1.1	43.6 ± 2.3
Triglyceride (mg/dL)	104.2 ± 1.4	131.1 ± 2.2	143.5 ± 2.7	147.5 ± 2.5	139.0 ± 1.6
HDL-cholesterol (mg/dL)	51.6 ± 0.2	51.1 ± 0.2	50.6 ± 0.2	50.3 ± 0.2	50.3 ± 0.2
LDL-cholesterol (mg/dL)	106.6 ± 1.2	119.1 ± 1.4	119.2 ± 1.6	116.3 ± 1.2	109.4 ± 1.1
25-vitamin D (ng/mL)	15.6 ± 0.2	16.0 ± 0.2	17.0 ± 0.2	17.8 ± 0.2	18.4 ± 02
UACR (mg/g Cr)	9.4 ± 1.6	10.9 ± 1.9	14.1 ± 1.8	20.3 ± 2.5	29.4 ± 2.5
Non-invasive indices of NAFLD					
AST/ALT ratio	1.23 ± 0.01	1.12 ± 0.01	1.14 ± 0.01	1.19 ± 0.01	1.38 ± 0.01
Age/PLT Index	0.41 ± 0.01	1.14 ± 0.02	2.30 ± 0.02	3.27 ± 0.02	5.01 ± 0.02
APRI	0.165 ± 0.001	0.191 ± 0.002	0.207 ± 0.002	0.231 ± 0.002	0.351 ± 0.005
BARD score	0.94 ± 0.01	0.99 ± 0.01	1.11 ± 0.01	1.29 ± 0.01	1.42 ± 0.01
Fib-4	0.350 ± 0.002	0.587 ± 0.001	0.775 ± 0.001	1.040 ± 0.002	1.789 ± 0.011
Fibrometer	16.41 ± 0.03	17.98 ± 0.03	18.84 ± 0.03	19.78 ± 0.03	21.34 ± 0.03
FLI^a^	17.6 ± 0.8	27.4 ± 1.2	29.7 ± 1.1	29.6 ± 0.8	29.5 ± 0.81
Forns index^a^	3.92 ± 0.04	5.10 ± 0.04	6.00 ± 0.04	6.71 ± 0.03	7.94 ± 0.03
Gholam’s model	7.91 ± 0.02	7.90 ± 0.02	8.07 ± 0.02	8.23 ± 0.02	8.50 ± 0.02
HSI	33.4 ± 0.1	33.7 ± 0.1	34.0 ± 0.1	34.7 ± 0.1	36.3 ± 0.1
ZJU index	33.4 ± 0.1	34.7 ± 0.1	35.2 ± 0.1	35.7 ± 0.1	36.2 ± 0.1

**Notes.**

The result is expressed as mean ± SD or frequencies (and proportions).

a Applicable only to 2011.

b Estimated using the Chronic Kidney Disease Epidemiology Collaboration (CKD-EPI) equation.

BP blood pressure eGFR estimated glomerular filtration rate HDL high-density lipoprotein LDL low-density lipoprotein UACR Urine albumin/Cr ratio Cr creatinine NAFLD non-alcoholic fatty liver disease AST Aspartate aminotransferase ALT Alanine aminotransferase APRI AST to PLT ratio index Fib-4 fibrosis-4 FLI fatty liver index Gholam’s model Gholam’s model for non-alcoholic steatohepatitis HSI hepatic steatosis index

### Relation of NAFLD with impaired kidney function and related risk factors

**Figure 1:**
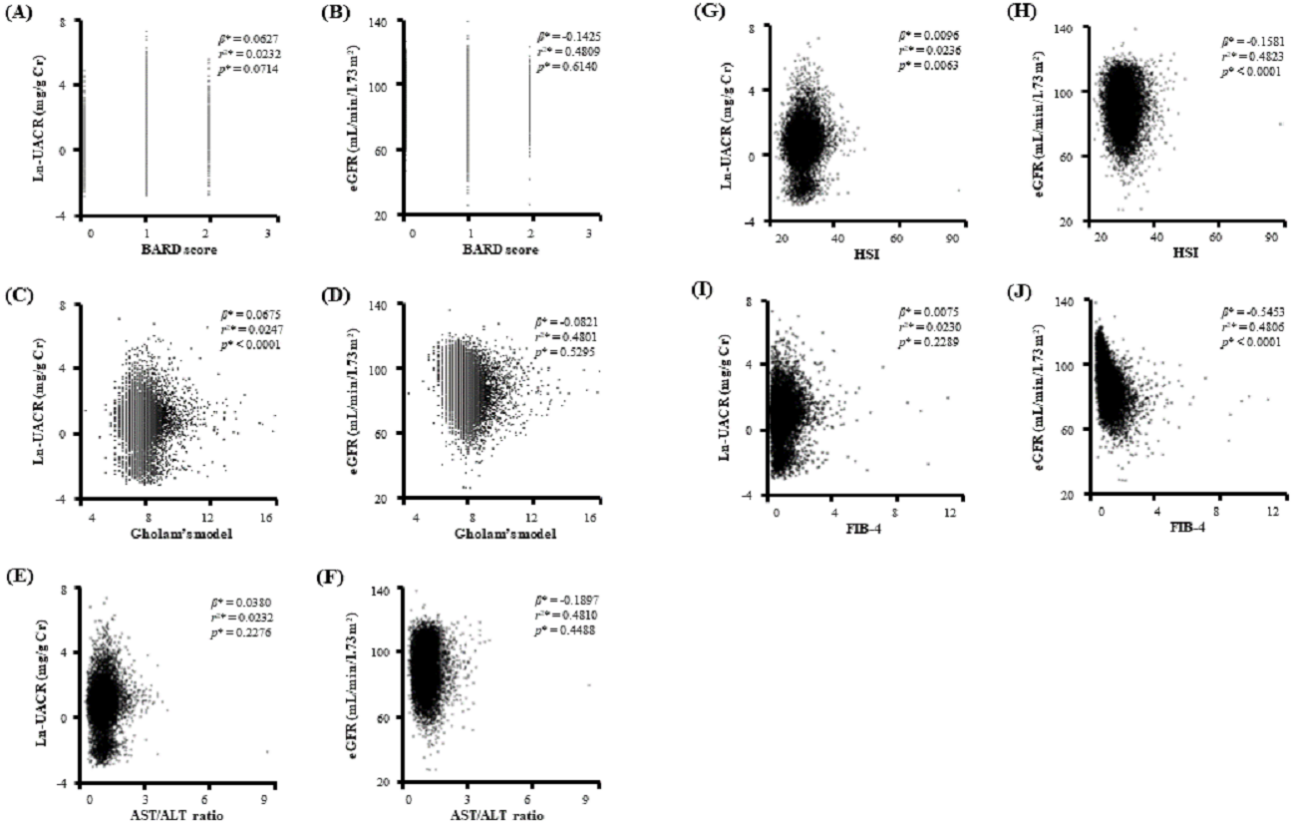
Scatter plots showing the relationship of candidate indices of hepatic fibrosis with Ln-UACR and eGFR. *Calculated by linear regression analysis using age, sex, and smoking as covariates Ln-UACR, logarithmic transformed urine albumin/creatinine ratio; eGFR, estimated glomerular filtration rate; Gholam’s model for non-alcoholic steatohepatitis; AST, aspartate aminotransferase; ALT, Alanine aminotransferase; HSI, hepatic steatosis index; FIB-4, fibrosis-4.

We performed a linear regression analysis with age, sex, and smoking history as covariates to find the possible relation of NAFLD with other clinical characteristics related to systemic metabolic disturbance using FIB-4 as candidate non-invasive index for NAFLD. As shown in the Table 2, FIB-4 was related with important components of metabolic syndrome (MetS) and kidney dysfunction. Interestingly, our scatter plot analysis indicated that there was some difference in relationship to kidney function between non-invasive indices (Fig. 1). While Gholam’s model for non-alcoholic steatohepatitis (*β* = 0.0675, *r*^2^ = 0.0247, *p* < 0.0001) had relation with Ln-UACR, hepatic steatosis index (HSI, *β* =  − 0.1581, *r*^2^ = 0.4823, *p* < 0.0001) and FIB-4 (*β* =  − 0.5453, *r*^2^ = 0.4806, *p* < 0.0001) were more related to eGFR. However, BARD score and aspartate aminotransferase/alanine aminotransferase (AST/ALT) ratio were not significantly related to either Ln-UACR or eGFR.

**Table 2:** Linear regression for FIB-4.

Variable	Crude		Model I	
	Slope	*p*	Slope	*p*
Age (year)	0.0260	<0.0001		
Female (vs. male)	−0.0258	0.0106		
Smoker (vs. non-smoker)	0.0673	<0.0001		
Systolic BP (mmHg)	0.0073	<0.0001	0.0013	0.0127
Diastolic BP (mmHg)	0.0032	<0.0001	0.0030	<0.0001
Body mass index (kg/m^2^)	0.0062	<0.0001	0.0168	<0.0001
Waist circumference (cm)	0.0018	<0.0001	0.0065	<0.0001
White blood cell (10^9^/L)	−0.0609	<0.0001	−0.0466	<0.0001
Hemoglobin (g/dL)	−0.0133	<0.0001	−0.0024	0.6216
Platelet (10^3^/µL)	−0.0041	<0.0001	−0.0036	<0.0001
eGFR^a^ (mL/min/1.73 m^2^)	−0.0154	<0.0001	−0.0028	0.0100
Fasting plasma glucose (mg/dL)	0.0078	<0.0001	0.0042	<0.0001
Hemoglobin A1c (%)	0.2033	<0.0001	0.1933	0.0541
AST (U/L)	0.0156	<0.0001	0.0126	<0.0001
ALT (U/L)	0.0030	<0.0001	0.0028	<0.0001
*γ*-Glutamyl transferase (U/L)^a^	0.0026	<0.0001	0.0015	0.0011
Triglyceride (mg/dL)	0.0002	<0.0001	0.0003	<0.0001
HDL-cholesterol (mg/dL)	−0.0001	0.9512		
LDL-cholesterol (mg/dL)	0.0001	0.8365		
25-vitamin D (ng/mL)	0.0142	<0.0001	0.0001	0.9937
Ln-UACR (mg/g Cr)	0.0171	0.0002	0.0133	0.1080

**Notes.**

a Applicable only to 2011.

Model I, adjusted for age, sex, and smoking.

Ln-UACR logarithmic transformed urine albumin/creatinine ratio

### Association of NAFLD with impaired kidney function

In the first step of multiple logistic regression analyses to estimate the association of non-invasive indices with albuminuria, we found that HSI was an independent predictor of albuminuria (adjusted OR = 1.071, 95% CI [1.031–1.113], Table 3), even after adjustment for clinically important risk factors related to vascular endothelial dysfunction. In the second step to find independent predictors of kidney dysfunction, the crude OR of the FIB-4 for incipient CKD was 1.929 (95% CI [1.658–2.244]), and the adjustment for age, sex, smoking, systolic BP, white blood cell, platelet, fasting plasma glucose, and TG did not weaken this association (adjusted OR = 1.254, 95% CI [1.034–1.521], Table 4).

**Table 3:** Multivariable logistic regression for albuminuria.^a^

Variable	Crude		Model I		Model II		Model III	
	OR	95% CI	OR	95% CI	OR	95% CI	OR	95% CI
Age (year)	1.037	1.029–1.045						
Female (vs. male)	1.931	1.501–2.483						
Smoker (vs. non-smoker)	1.326	0.921–1.912						
Systolic BP (mmHg)	1.029	1.016–1.043	1.022	1.009–1.035				
Diastolic BP (mmHg)	1.011	0.995–1.027						
Body mass index (kg/m^2^)	1.037	0.990–1.086						
Waist circumference (cm)	1.011	0.995–1.026						
White blood cell (10^9^/L)	1.181	1.097–1.271	1.113	1.032–1.200				
Hemoglobin (g/dL)	0.855	0.822–0.959	0.935	0.810–1.081				
Platelet (10^3^/µL)	1.003	1.001–1.006	1.003	1.001–1.006				
Fasting plasma glucose (mg/dL)	1.035	1.020–1.050	1.027	1.012–1.042	1.021	1.006–1.036		
Hemoglobin A1c (%)	1.490	0.984–2.257						
Aspartate aminotransferase (U/L)	1.006	1.001–1.009	1.005	1.001–1.010	1.005	1.001–1.010	1.005	1.001–1.010
Alanine aminotransferase (U/L)	1.004	0.987–1.007						
*γ*-Glutamyl transferase (U/L)^b^	1.001	0.996–1.006						
Triglyceride (mg/dL)	1.001	1.001–1.002	1.001	1.000–1.002	1.001	0.999–1.002		
HDL-cholesterol (mg/dL)	0.995	0.983–1.006						
LDL-cholesterol (mg/dL)	1.011	1.003–1.020	1.008	0.999–1.017				
25-vitamin D (ng/mL)	1.018	0.980–1.046						
AST/ALT ratio	1.794	1.256–2.561	1.785	1.524–2.479	1.643	1.150–2.349	1.460	1.007–2.118
Age/PLT Index	1.242	1.229–1.325	1.027	0.906–1.163				
APRI	1.234	0.897–1.697						
BARD score	1.891	1.302–2.745	1.603	1.049–2.448	1.511	1.039–2.198	1.478	1.030–2.121
FIB-4	1.236	1.156–1.323	1.019	0.916–1.133				
Fibrometer	1.194	1.073–1.328	1.043	0.842–1.293				
FLI^b^	1.016	1.006–1.026	1.013	1.002–1.024	1.011	1.002–1.021	1.011	0.998–1.021
Forns index^b^	1.248	1.011–1.539	1.031	0.741–1.436				
Gholam’s model	1.210	1.075–1.362	1.203	1.057–1.370	1.201	1.054–1.369	1.186	1.039–1.353
HSI	1.106	1.076–1.137	1.080	1.040–1.121	1.072	1.032–1.113	1.071	1.031–1.113
ZJU index	1.104	1.067–1.142	1.082	1.039–1.127	1.058	1.016–1.101	1.046	1.003–1.092

**Notes.**

a Defined as an UACR of 30 mg/g Cr or more.

b Applicable only to 2011.

Model I, performed using age, sex, and smoking as covariates.

Model II, performed using age, sex, and smoking as covariates and systolic BP, white blood cell, and platelet as predictors.

Model III, performed using age, sex, and smoking as covariates and systolic BP, white blood cell, platelet, and fasting plasma glucose as predictors.

OR odds ratio CI confidence interval

**Table 4:** Multivariable logistic regression for chronic kidney disease^a^.

Variable	Crude		Model I		Model II		Model III	
	OR	95% CI	OR	95% CI	OR	95% CI	OR	95% CI
Age (year)	1.048	1.039–1.057						
Female (vs. male)	1.918	1.535–2.397						
Smoker (vs. non-smoker)	1.538	1.093–2.165						
Systolic BP (mmHg)	1.033	1.021–1.045	1.022	1.010–1.033				
Diastolic BP (mmHg)	1.001	0.987–1.015						
Body mass index (kg/m^2^)	1.026	0.981–1.072						
Waist circumference (cm)	1.008	0.994–1.023						
White blood cell (10^9^/L)	1.208	1.129–1.293	1.127	1.053–1.206				
Hemoglobin (g/dL)	0.855	0.800–0.913	0.988	0.876–1.114				
Platelet (10^3^/µL)	1.003	1.001–1.005	1.002	1.001–1.005				
Fasting plasma glucose (mg/dL)	1.032	1.019–1.046	1.023	1.009–1.036	1.017	1.003–1.030		
Hemoglobin A1c (%)	1.517	1.044–2.204	1.182	0.590–1.214				
Aspartate aminotransferase (U/L)	1.006	1.001–1.010	1.006	1.001–1.010	1.005	1.001–1.010	1.005	1.001–1.010
Alanine aminotransferase (U/L)	1.003	0.988–1.007						
*γ*-Glutamyl transferase (U/L)^b^	1.001	0.991–1.007						
Triglyceride (mg/dL)	1.002	1.001–1.002	1.002	1.001–1.002	1.001	1.001–1.002		
HDL-cholesterol (mg/dL)	0.994	0.984–1.005						
LDL-cholesterol (mg/dL)	1.011	1.002–1.020	1.007	0.998–1.017				
25-vitamin D (ng/mL)	1.011	0.988–1.036						
AST/ALT ratio	1.981	1.443–2.718	1.944	1.524–2.479	1.783	1.291–2.462	1.543	1.103–2.160
Age/PLT Index	1.306	1.229–1.389	1.032	0.925–1.152				
APRI	1.296	0.986–1.703						
BARD score	1.952	1.415–2.693	1.652	1.135–2.404	1.575	1.129–2.197	1.548	1.122–2.135
FIB-4	1.929	1.658–2.244	1.814	1.425–2.309	1.785	1.393–2.288	1.254	1.034–1.521
Fibrometer	1.273	1.156–1.402	1.065	0.904–1.253				
FLI^b^	1.018	1.009–1.027	1.013	1.004–1.023	1.010	1.002–1.018	1.008	0.996–1.021
Forns index^b^	1.287	1.060–1.562	1.031	0.771–1.380				
Gholam’s model	1.228	1.100–1.372	1.201	1.052–1.371	1.197	1.049–1.365	1.185	1.038–1.353
HSI	1.109	1.080–1.138	1.079	1.041–1.117	1.073	1.036–1.111	1.072	1.034–1.111
ZJU index	1.104	1.069–1.141	1.080	1.039–1.124	1.056	1.016–1.111	1.044	0.996–1.093

**Notes.**

a Defined as an eGFR less than 60 mL/min/1.73 m^2^ and UACR of 30 mg/g Cr or more.

b Applicable only to 2011.

Model I, performed using age, sex, and smoking as covariates.

Model II, performed using age, sex, and smoking as covariates and systolic BP, white blood cell, and platelet as predictors.

Model III, performed using age, sex, and smoking as covariates and systolic BP, white blood cell, platelet, fasting plasma glucose, and triglyceride as predictors.

OR odds ratio CI confidence interval

**Figure 2:**
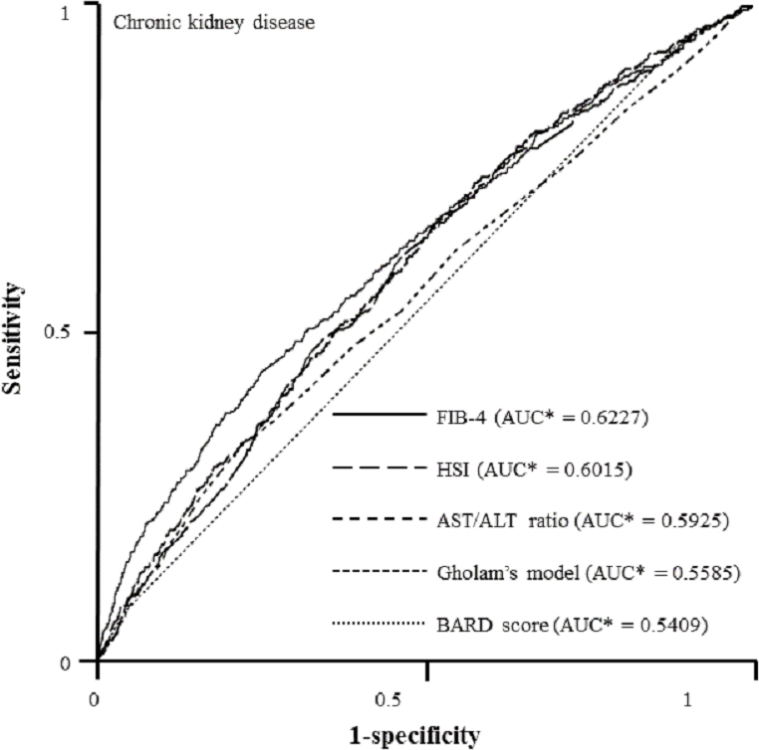
ROC curves representing the prediction capacity of risk for chronic kidney disease***. As compared with AST/ALT ratio (AUC* = 0.5925, 95% CI* [0.5655–0.6195]), FIB-4 had best predictive value for CKD (FIB-4, AUC* = 0.6227, 95% CI* [0.5943–0.6511], p** = 0.0256; HSI, AUC* = 0.6015, 95% CI* [0.5746–0.6284], p** = 0.5426; Gholam’s model for non-alcoholic steatohepatitis, AUC* = 0.5585, 95% CI* [0.5296–0.5875], p** = 0.1089; BARD score, AUC* = 0.5409, 95% CI* [0.5257–0.5562], p** = 0.0004). *Calculated by logistic regression analysis using age, sex, and smoking as covariates and systolic BP, white blood cell, platelet, fasting plasma glucose, aspartate aminotransferase, and triglyceride as predictors. **Estimated using nonparametric methods previously described by DeLong, et. al. ***Defined as an eGFR less than 60 mL/min/1.73 m^2^ and UACR of 30 mg/g Cr or more. ROC, Receiver-operating characteristic; AUC, areas under the ROC curves; CI, confidence interval.

In the final step to compare the predictive capacities of non-invasive indices for incipient CKD, we created ROC curves by performing logistic regression model using age, sex, and smoking as covariates and systolic BP, white blood cell, platelet, fasting plasma glucose, TG, and AST as predictors (Fig. 2). FIB-4 had the best predictive value for CKD (FIB-4, AUC = 0.6227, 95% CI [0.5943–0.6511], *p* = 0.0256; HSI, AUC = 0.6015, 95% CI [0.5746–0.6284], *p* = 0.5426; Gholam’s model for non-alcoholic steatohepatitis, AUC = 0.5585, 95% CI [0.5296–0.5875], *p* = 0.1089; BARD score, AUC = 0.5409, 95% CI [0.5257–0.5562], *p* = 0.0004) as compared with AST/ALT ratio (AUC = 0.5925, 95% CI [0.5655–0.6195]). However, there was no significant difference between FIB-4 and HSI (*Z* value for comparison among ROC: FIB-4—HIS, 1.247; FIB-4—Gholam’s model, 3.567; FIB-4—BARD score, 5.382; FIB-4—AST/ALT ratio, 2.221).

## Discussion

This study provides an in-depth overview of the diagnostic performance of non-invasive indices of NAFLD to detect extra-hepatic effect on the kidney, showing that non-invasive indices of NAFLD closely associated with clinically important components of MetS and FIB-4 had best predictive ability to identify increased risk of incipient CKD in the general population. These findings suggest that hepatic necro-inflammation intricately related to incipient CKD and FIB-4 could be value in describing these pathologic links.

In this study, participants in the highest FIB-4 quintile were more obese and there was a close relationship between FIB-4 and clinical parameters related with insulin resistance and systemic inflammation. The liver plays a major role in glucose and lipid metabolism in our body. In patients with MetS, hepatic necro-inflammation may have a potent negative impact on lipid metabolism, the innate and acquired immune systems, coagulation pathway, and vascular endothelium via activation of various pro-inflammatory and pro-fibrotic signaling pathways (Paquissi, 2016; Pasarin et al., 2012; Zhang et al., 2015). Such pathologic responses contribute to the development of vascular endothelial dysfunction and increased risk of various target organ damages, including cardiovascular disease and CKD (Choi et al., 2017; Kim et al., 2012). Our results provide clinical evidence that potential communication and cross-talk between abnormal lipid uptake and systemic inflammatory responses can be a regulator of the development of target organ damage.

Our scatter plot and logistic regression analysis showed that there is some difference in discrimination power of candidate non-invasive indices to distinguish possible proteinuric kidney injury or renal excretory dysfunction from the healthy state. Pathologic connection of kidney injury-interstitial inflammation-renal fibrogenesis is generally accepted as a key feature of progressive renal injury (Meng, Nikolic-Paterson & Lan, 2014). However, some authors argued that the fate-determining mechanisms that affect vascular endothelium and kidney functions may be different from phase to phase (Kurts et al., 2013; Liu, 2011; Ueha, Shand & Matsushima, 2012; Weber, 2004; Wynn, 2007). In an exaggerated inflammation state, circulatory pro-inflammatory mediators can alter glomerular filtration barrier function and increase protein excretion into tubular lumen, which triggers a cascade of tubulointerstitial inflammation (Lee & Kalluri, 2010). In addition to proteinuric kidney injury, hemodynamic instability and hypoxic renal injury in patients with NAFLD contribute to more extensive renal fibrosis and progressive loss of kidney function (Guerrot et al., 2012). To prevent the progression of kidney disease, the discrimination between initiation of renal injury and progression of tubulointerstitial inflammation is important in managing patients with kidney disease (Hodgkins & Schnaper, 2012; Parikh & Mansour, 2017). In this regard, proper combination of candidate non-invasive indices could help monitor kidney disease progression by differentiation between renal injury initiation and more extensive tubulointerstitial inflammation, which can alter treatment strategies to reduce the risk of CKD and end-stage renal disease.

Our logistic regression analysis revealed that some non-invasive indices were independently associated with impaired kidney function in the general population. Previous clinical studies showed that increased non-invasive indices of NAFLD, such as fatty liver index and FIB-4, had a significant relationship with CKD defined by eGFR, but they did not demonstrate whether minimal hepatic necro-inflammation, which could not be observed by ultrasound, relates to initiation of systemic inflammation and development of kidney damage presenting with microalbuminuria preceding the decrease in eGFR (Huh et al., 2017; Wijarnpreecha et al., 2017; Xu et al., 2016). Our results suggest that systemic effects of dysfunctional hepatic lipid uptake on kidney function may be prominent, even before the appearance of overt CKD or NAFLD, and FIB-4 and some non-invasive indices could be valuable for predicting the initiation of this pathologic communication.

In our ROC curve analysis, we found that FIB-4 and HSI had better precision in estimating the risk of incipient CKD. However, the AUC values of candidate non-invasive indices were lower than that in other previous reports (Huh et al., 2017; Xu et al., 2016). One possible explanation is that the difference in study population between our study and previous studies may be a cause of lower frequency of NAFLD and related extrahepatic manifestations. Another explanation is that because conventional non-invasive methods were originally designed to identify subjects at risk of severe hepatic steatosis and advanced liver fibrosis, they may have possible limitations in the early detection of extrahepatic organ injury, especially the kidney. Thus, there remains a compelling need of more reliable diagnostic tools to assess pathologic processes of kidney injury or pharmacologic responses to therapeutic interventions.

This study possessed several limitations. First, we did not have radiologic and pathologic data concerning hepatic steatosis. Thus, a positive control with NAFLD and a negative control without NAFLD were not included in the study because no liver biopsy was performed. Serum liver function tests (LFTs) are not completely sensitive for NAFLD diagnosis and the full spectrum of histopathologic findings could be observed in subjects with normal LFT results (Anstee, Targher & Day, 2013; Chalasani et al., 2012). However, recently designed non-invasive methods have improved precision in differentiating steatohepatitis form simple steatosis without liver biopsy and some of them have adequate diagnostic performance to substitute for radiologic and pathologic studies (Buzzetti et al., 2015; Dyson, Anstee & McPherson, 2014; Pearce, Thosani & Pan, 2013). Such findings suggest that the novel indices of NAFLD used in this study may be useful to predict early liver necro-inflammation as well as indicate its related distant organ dysfunctions before an increase in LFT levels in healthy patients and also those metabolic syndrome and no identified steatohepatitis. Second, the initiation and progression of kidney disease are influenced by various factors. Owing to the limitations of our study design, we could not adjust for various factors other than age, sex, smoking history, systolic BP, white blood cell, platelet, fasting plasma glucose, AST, and TG. Third, because of the self-reporting of medication, medical history, and use of alcohol and tobacco, a social-desirability bias cannot be excluded. It may be responsible for the discrepancy between present experiments and previous research. Finally, participants may have forgotten certain relevant details.

In conclusion, our results indicate that there is a close relation between hepatic necro-inflammation and impaired kidney function and FIB-4 may be superior in determining the risk of metabolic disturbance and its related kidney dysfunction in the general population. A large, population-based, prospective, epidemiologic study is warranted to confirm these findings.

##  Supplemental Information

10.7717/peerj.6524/supp-1 Figure S1 Flow chart of the study group enrolment process

10.7717/peerj.6524/supp-2 Table S1 Non-invasive indices of hepatic fibrosis *Applicable only to 2011. AST (U/L), Aspartate aminotransferase; ALT (U/L), Alanine aminotransferase; APRI, AST to PLT ratio index; BMI (kg/m^2^), body mass index; DM, diabetes mellitus; FIB-4, fibrosis-4; FPG (mg/dL), fasting plasma glucose; FLI, fatty liver index; Gholam’s model, Gholam’s model for non-alcoholic steatohepatitis; HSI, hepatic steatosis index; GGT (U/L), *γ*-Glutamyl transferase; Ln, Natural logarithm; PLT (10^9^/L), Platelet; TG (mg/dL), triglyceride; WC (cm), waist circumference; Wt (kg), weight.

## Additional Information and Declarations

### Data Availability

The following information was supplied regarding data availability: The research in this article did not generate any data or code. Data was retrieved from https://knhanes.cdc.go.kr/knhanes/eng/index.do.
