# Supplementary figures and images for: Comparison of laboratory indices of non-alcoholic fatty liver disease for the detection of incipient kidney dysfunction
Source: PeerJ. 2019 Mar 8;7:e6524. doi: 10.7717/peerj.6524 (PMC6410686; doi:10.7717/peerj.6524)

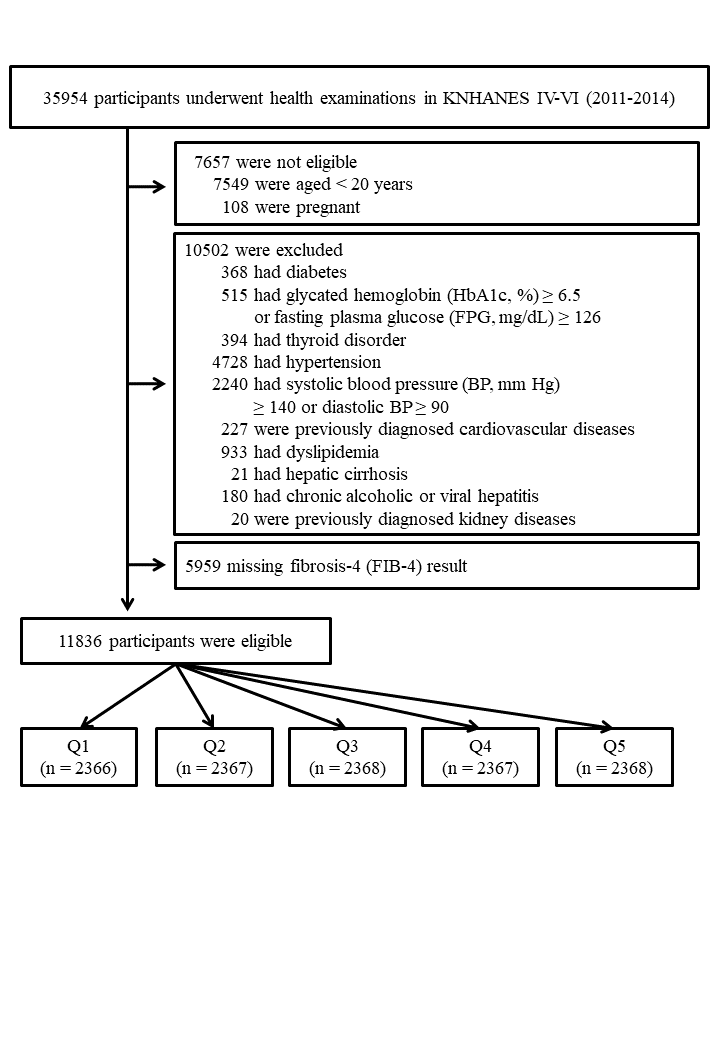

Supplement: Figure S1 [file peerj-07-6524-s001.png]
