# Supplementary material for: Comparison of laboratory indices of non-alcoholic fatty liver disease for the detection of incipient kidney dysfunction
Source: PeerJ. 2019 Mar 8;7:e6524. doi: 10.7717/peerj.6524 (PMC6410686; doi:10.7717/peerj.6524)
Supplement: Table S1 — *Applicable only to 2011. AST (U/L), Aspartate aminotransferase; ALT (U/L), Alanine aminotransferase; APRI, AST to PLT ratio index; BMI (kg/m2), body mass index; DM, diabetes mellitus; FIB-4, fibrosis-4; FPG (mg/dL), fasting plasma glucose; FLI, fatty liver index; Gholam’s model, Gholam’s model for non-alcoholic steatohepatitis; HSI, hepatic steatosis index; GGT (U/L), γ-Glutamyl transferase; Ln, Natural logarithm; PLT (109/L), Platelet; TG (mg/dL), triglyceride; WC (cm), waist circumference; Wt (kg), weight. [file peerj-07-6524-s002.docx]

Supplementary Table S1. Non-invasive indices of hepatic fibrosis

| Method | Formula for hepatic fibrosis | Reference |
| --- | --- | --- |
| AST/ALT ratio | AST / ALT. | ^(^*^Abdelgawad 2015^*^)^ |
| Age/PLT Index | Age score + PLT score.  Age (year): < 30 = 0, 30-39 = 1, 40-49 = 2, 50-59 = 3, 60-69 = 4, ≥ 70 = 5.  PLT count: ≥ 225 = 0, 200-224 = 1, 175-199 = 2, 150-174 = 3, 125-149 = 4, < 125 = 5. | ^(^*^Abdelgawad 2015^*^)^ |
| APRI | [AST / upper normal limit] / PLT x 100. | ^(^*^Wai et al. 2003^*^)^ |
| BARD score | BMI score + AST/ALT ratio score + DM score  BMI: < 28 = 0, ≥ 28 = 1.  AST/ALT ratio: < 0.8 = 0, ≥ 0.8 = 1.  DM: non-diabetic =0, type 2 DM =1. | ^(^*^Harrison et al. 2008^*^)^ |
| FIB-4 | [Age (year) x AST] / [PLT x AST^1/2^]. | ^(^*^Sterling et al. 2006^*^)^ |
| Fibrometer | 0.4184 x FPG (mmol/L) + 0.0701 x AST + 0.00008 ferritin (mg/L) – 0.0102 x PLT – 0.0260 x ALT + 0.0459 x Wt + 0.0842 x Age (year) + 11.6226. | ^(^*^Cales et al. 2005^*^)^ |
| FLI* | e^0.953 × Ln (TG) + 0.139 × BMI + 0.718 × Ln (GGT*) + 0.053 × WC - 15.745^/(1+e^0.953 × Ln (TG) + 0.139 × BMI + 0.718 × Ln (GGT*) + 0.053 × WC - 15.745^) × 100 | ^(^*^Choi et al. 2017^*^)^ |
| Forns index* | 7.811 – 3.131 x Ln (PLT) + 0.781 x Ln (GGT*) + 3.467 x Ln [age (year)] – 0.014 * cholesterol. | ^(^*^Forns et al. 2002^*^)^ |
| Gholam’s model | 2.627 x Ln (AST) + 2.13 if DM. | ^(^*^Gholam et al. 2007^*^)^ |
| HSI | 8 x AST/ALT ratio + BMI (+ 2, if DM; + 2, if female) | ^(^*^Lee et al. 2010^*^)^ |
| ZJU index | BMI (kg/m^2^) + FPG (mmol/L) + TG (mmol/L) + 3 x AST (U/L) / ALT (U/L) (+2, in female). | ^(^*^Poynard et al. 2004^*^)^ |

*applicable only to 2011.

AST (U/L), Aspartate aminotransferase; ALT (U/L), Alanine aminotransferase; APRI, AST to PLT ratio index; BMI (kg/m^2^), body mass index; DM, diabetes mellitus; FIB-4, fibrosis-4; FPG (mg/dL), fasting plasma glucose; FLI, fatty liver index; Gholam’s model, Gholam’s model for non-alcoholic steatohepatitis; HSI, hepatic steatosis index; GGT (U/L), γ-Glutamyl transferase; Ln, Natural logarithm; PLT (10^9^/L), Platelet; TG (mg/dL), triglyceride; WC (cm), waist circumference; Wt (kg), weight.

Abdelgawad IA. 2015. Clinical utility of simple non-invasive liver fibrosis indices for predicting hepatocellular carcinoma (HCC) among Egyptian patients. *J Clin Pathol* 68:154-160. 10.1136/jclinpath-2014-202462

Cales P, Oberti F, Michalak S, Hubert-Fouchard I, Rousselet MC, Konate A, Gallois Y, Ternisien C, Chevailler A, and Lunel F. 2005. A novel panel of blood markers to assess the degree of liver fibrosis. *Hepatology* 42:1373-1381. 10.1002/hep.20935

Choi JW, Oh IH, Lee CH, and Park JS. 2017. Is there a J-shaped relationship between the fatty liver index and risk of microalbuminuria in the general population? *Clin Chim Acta*. 10.1016/j.cca.2017.08.015

Forns X, Ampurdanes S, Llovet JM, Aponte J, Quinto L, Martinez-Bauer E, Bruguera M, Sanchez-Tapias JM, and Rodes J. 2002. Identification of chronic hepatitis C patients without hepatic fibrosis by a simple predictive model. *Hepatology* 36:986-992. 10.1053/jhep.2002.36128

Gholam PM, Flancbaum L, Machan JT, Charney DA, and Kotler DP. 2007. Nonalcoholic fatty liver disease in severely obese subjects. *Am J Gastroenterol* 102:399-408. 10.1111/j.1572-0241.2006.01041.x

Harrison SA, Oliver D, Arnold HL, Gogia S, and Neuschwander-Tetri BA. 2008. Development and validation of a simple NAFLD clinical scoring system for identifying patients without advanced disease. *Gut* 57:1441-1447. 10.1136/gut.2007.146019

Lee JH, Kim D, Kim HJ, Lee CH, Yang JI, Kim W, Kim YJ, Yoon JH, Cho SH, Sung MW, and Lee HS. 2010. Hepatic steatosis index: a simple screening tool reflecting nonalcoholic fatty liver disease. *Dig Liver Dis* 42:503-508. 10.1016/j.dld.2009.08.002

Poynard T, Munteanu M, Imbert-Bismut F, Charlotte F, Thabut D, Le Calvez S, Messous D, Thibault V, Benhamou Y, Moussalli J, and Ratziu V. 2004. Prospective analysis of discordant results between biochemical markers and biopsy in patients with chronic hepatitis C. *Clin Chem* 50:1344-1355. 10.1373/clinchem.2004.032227

Sterling RK, Lissen E, Clumeck N, Sola R, Correa MC, Montaner J, M SS, Torriani FJ, Dieterich DT, Thomas DL, Messinger D, and Nelson M. 2006. Development of a simple noninvasive index to predict significant fibrosis in patients with HIV/HCV coinfection. *Hepatology* 43:1317-1325. 10.1002/hep.21178

Wai CT, Greenson JK, Fontana RJ, Kalbfleisch JD, Marrero JA, Conjeevaram HS, and Lok AS. 2003. A simple noninvasive index can predict both significant fibrosis and cirrhosis in patients with chronic hepatitis C. *Hepatology* 38:518-526. 10.1053/jhep.2003.50346
